# Internal Malignancy Risk After Carbon Monoxide Poisoning: A Nationwide Population-Based Cohort Study

**DOI:** 10.3390/jcm14030937

**Published:** 2025-01-31

**Authors:** Gyo Jin Ahn, Solam Lee, Seok Jeong Lee, Yong Sung Cha

**Affiliations:** 1 Department of Emergency Medicine, Yonsei University Wonju College of Medicine, Wonju 26426, Republic of Korea; minstrel@yonsei.ac.kr; 2 Department of Dermatology, Yonsei University Wonju College of Medicine, Wonju 26426, Republic of Korea; solam@yonsei.ac.kr; 3 Division of Pulmonary, Allergy, and Critical Care Medicine, Department of Internal Medicine, Yonsei University Wonju College of Medicine, Wonju 26426, Republic of Korea; 4 Research Institute of Hyperbaric Medicine and Science, Yonsei University Wonju College of Medicine, Wonju 26426, Republic of Korea

**Keywords:** carbon monoxide, epidemiology, malignancy, poisoning, prognosis

## Abstract

**Background:** We aimed to investigate the association between acute carbon monoxide (CO) poisoning and the risk of internal malignancies, including hematologic malignancies. **Methods:** The study population was derived from the National Health Insurance Service (NHIS) database of Korea between 2002 and 2022. Adults diagnosed with CO poisoning and controls were included. Demographics, socioeconomic statuses, lifestyle factors, and comorbidity profiles of participants were retrieved from the NHIS database. Covariates potentially associated with disease outcomes were selected based on the available literature and biological plausibility, balanced between the two cohorts using inverse probability of treatment weighting, and applied to adjust multivariable models. **Results:** Overall, 42,874 patients with CO poisoning and 905,285 controls were included; both cohorts comprised 44.3% females. The mean age of the CO poisoning and controls was 51.5 and 50.9 years, respectively. Patients with CO poisoning had a 1.02-fold increase in the overall risk of malignancy (a 1.03-fold increase in solid organ malignancies and a 0.71-fold decrease in hematologic malignancies) compared with controls. The risk of internal malignancy was increased in the oral cavity (adjusted hazard ratio, 1.33; 95% confidence intervals, 1.19–1.49), lungs (1.39; 1.33–1.46), bone (1.68; 1.23–2.30), cervix (1.32; 95% CI, 1.17–1.49), and kidneys (1.14; 1.04–1.24). Conversely, the risk of internal malignancies was decreased in the thorax (0.59; 0.45–0.77), anus (0.14; 0.06–0.34), uterus (0.71; 0.60–0.82), ovaries (0.59; 0.45–0.77), prostate (0.89; 0.84–0.95), Hodgkin lymphoma (0.35; 0.20–0.61), non-Hodgkin lymphoma (0.67; 0.59–0.75), and multiple myeloma (0.36; 0.30–0.43). **Conclusions:** CO poisoning was associated with the development of internal malignancies.

## 1. Introduction

Approximately 50,000 individuals in the United States are admitted to emergency departments annually owing to carbon monoxide (CO) poisoning, resulting in an estimated 1500 deaths [1,2,3]. Notably, 25–50% of acute CO poisoning survivors [4] develop neurocognitive sequelae [5]. Additionally, CO poisoning can cause heart [6,7] and kidney damage [8,9], with those experiencing cardiac injury facing a particularly high risk of mortality [10]. Furthermore, the long-term consequences of CO poisoning extend beyond neurocognitive and cardiovascular complications. In our recent study, the CO poisoning cohort had a substantially higher risk of long-term mortality owing to various diseases than the control cohort [11].

Based on GLOBOCAN 2020 data [12], approximately 19 million new cancer cases and 10 million cancer-related deaths were reported globally in 2020, making cancer a leading cause of morbidity and mortality worldwide. Furthermore, this trend is expected to intensify, as projections indicate that the global incidence of cancer will rise from 19.3 million cases in 2020 to 28.4 million by 2040, reflecting a 47% increase. This surge in incidence is also anticipated to be accompanied by a corresponding rise in cancer-related mortality [12].

Recent studies have demonstrated that low-dose CO can exert antiapoptotic, anti-inflammatory, and antioxidant properties, in addition to vasodilatory and antiproliferative effects that promote tissue regeneration [13]. Several experimental and preclinical models have shown that enhancing endogenous CO production or delivering exogenous CO may have therapeutic potential in various diseases, including malignancies [13,14]. However, it is crucial to recognize that the therapeutic application of low CO concentrations differs considerably from high concentrations associated with CO poisoning. Currently, the potential impact of CO poisoning on the long-term development of malignancies in humans remains unclear.

Therefore, in this study, we aimed to investigate the risk of internal malignancies, including hematologic malignancies, in patients with CO poisoning using nationwide, population-based cohort data.

## 2. Materials and Methods

### 2.1. Data Source and Ethics Statement

In this nationwide population-based cohort study, data were obtained from the National Health Insurance Service (NHIS) administrative database in Korea, covering nearly 97% of the Korean population [15]. The medical aid program, which supports approximately 3% of the entire Korean population with the low-income population, has been incorporated into the NHIS, resulting in comprehensive coverage of nearly the entire Korean population. Individuals enrolled in the NHIS are required to undergo biennial health examinations, and the results are systematically recorded in the general health examination database. These examinations are available to all employees, household heads, and individuals aged over 40 years. Consequently, since 2002, the NHIS has maintained an extensive database containing demographic information, socioeconomic data, diagnoses, and prescription records for over 50 million individuals.

This study was approved by the Korean National Institute for Bioethics Policy (approval number: NHIS-2022-1-366) and the institutional review board of Wonju Severance Christian Hospital (CR321347; Effects of Carbon Monoxide Poisoning on Prognosis, 2023-07-25). Additionally, the study was carried out in compliance with the ethical principles established in the 1975 Declaration of Helsinki. Given that the data were anonymized, the requirement for informed consent was waived.

### 2.2. Study Population

The study cohort was extracted from the NHIS database between 1 January 2002 and 31 December 2022. All individuals aged ≥18 years in the database were considered for inclusion. However, individuals diagnosed with any form of cancer between 2002 and 2003 were excluded based on the assumption that those who had not sought medical care for cancer during the 2-year period (2-year washout period) were no longer affected by the disease [16]. We defined patients with acute CO poisoning as those who had a history of medical facility visits recorded with T58 in the International Statistical Classification of Diseases, 10th revision (ICD-10), at least once between 2004 and 2022 and had no prior diagnosis of any cancer prior to the diagnosis of CO poisoning. The control group included individuals with no recorded ICD-10 code of T58 during the entire observation period, who were randomly assigned an index date reflecting the distribution of diagnosis dates of the CO poisoning group, and who had no prior diagnosis of any cancer as of the index date. The date of the first documented visit for CO poisoning was defined as the index date for each patient. If a patient experienced multiple visits for CO poisoning, only the first visit was included in the analysis. For control participants, the index date was the same as that of their matched cases. Follow-up started on the index date and continued until death, emigration, or the end of the observation period (31 December 2022), whichever came first.

### 2.3. Validation of Patient Identification with CO Poisoning Using ICD-10 Codes

The reliability of our criteria for identifying CO poisoning cases from the administrative database was initially assessed by reviewing the electronic medical records of patients who presented to Wonju Severance Christian Hospital between January 2006 and August 2022. A prospective CO poisoning registry, which included the CO poisoning cohort, was established with informed consent as part of the CARE CO cohort (ClinicalTrials.gov identifier: NCT04490317). Data were meticulously collected and managed by two clinical toxicology specialists from 2006 onward, minimizing the likelihood of errors owing to the performance of regular reviews.

At our institution, acute CO poisoning was diagnosed as based on the patient’s clinical history and elevated carboxyhemoglobin levels, with thresholds set at over 5% for non-smokers and over 10% for smokers. In cases where carboxyhemoglobin levels were within normal limits but the source of CO exposure was evident and the patient exhibited CO poisoning-related symptoms, a diagnosis of CO poisoning was established. We cross-referenced our CO poisoning registry with the T58 ICD-10 code for CO poisoning in hospital records over the same period and excluded cases of chronic CO poisoning from our analysis. Two board-certified emergency medicine specialists independently reviewed each case to validate the diagnosis and calculate the positive predictive value (PPV).

### 2.4. Outcome Measurement

The outcome was the incidence of solid organ and hematologic malignancies in patients with CO poisoning and matched controls. Malignancies of individual organ origin were separately investigated, as previously described [16]. The predefined outcomes and corresponding ICD-10 codes are summarized in Supplementary Table S1.

In Korea, the NHIS operates the Rare and Intractable Disease (RID) registration program, which gives patients with oncological diseases a 90% copayment reduction for treatment. For registration, all candidates must be assessed by a board-certified expert according to uniform standards established by the government. The institution reviews RID program applications of all patients, along with their diagnosis, to confirm that diagnostic criteria are met. Subsequently, the claims are forwarded to the NHIS, which includes them in the claims database. This systematic process ensures the reliability of the diagnosis of malignancies.

### 2.5. Covariates

The demographics, socioeconomic statuses, lifestyle factors, and comorbidity profiles of the study population were retrieved from the NHIS database. Covariables that could potentially be related to the disease outcome were set based on the previous literature and the biological plausibility of associations [14,17]. Age at the study index date, sex, insurance type (standard vs. medical aid), income level (divided into quartiles based on health insurance premiums), and the area of residence (urban vs. rural area) were determined. In the general health examination data, the current smoking status was established, and drinking was defined as routine alcohol consumption regardless of the amount or frequency. Additionally, the height, weight, waist circumference, systolic and diastolic blood pressure, serum levels of hemoglobin, fasting blood glucose, liver enzymes (aspartate aminotransferase, alanine transaminase), and creatinine levels at index date were recorded. Comorbidities, including diabetes (ICD-10: E10-14), hypertension (I10), dyslipidemia (E78), chronic obstructive pulmonary disease (J44), chronic kidney disease (N18), liver cirrhosis (K74, K703), and chronic heart failure (I50), were selected as confounding variables based on their association with the incidence of internal malignancies [18]. Additionally, the Charlson comorbidity index, which predicts outcomes like mortality depending on the severity of many comorbid conditions, was calculated for the index date [19]. The covariates were balanced between the two cohorts using inverse probability of treatment weighting (IPTW) and then applied to further adjust the multivariable models [20].

### 2.6. Statistical Analyses

The propensity scores for individuals were estimated as the probability of belonging to the CO poisoning cohort based on the covariates and were used to calculate the inverse probability weights, which were determined as follows: the probability of belonging to the CO poisoning cohort/(1 − the probability of belonging to the CO poisoning cohort). Covariate balances before and after the application of these weights were assessed using standardized mean differences. Subsequently, the risks of predefined outcomes were estimated for the CO poisoning cohort compared with those of the control cohort. Statistical estimates were derived using multivariable Cox proportional hazards analysis after adjusting for all covariates used in the inverse probability weighting. For each analysis, individuals diagnosed with the target outcome on or prior to the index date were excluded, ensuring that only those at risk were included in the analysis. Additionally, to verify the proportional hazards assumption for the Cox analysis, we assessed Schoenfeld residuals and confirmed the Schoenfeld *p*-values. To comprehensively investigate specific populations within both groups, subgroup analyses were conducted according to age, sex, administration of hyperbaric oxygen (HBO_2_) therapy, and intensive care unit (ICU) admission. Data analyses were performed using SAS statistical software (version 9.4; SAS Institute) and R statistical software (version 3.4.1; R Project for Statistical Computing), with the significance threshold set at 5%.

## 3. Results

### 3.1. Criteria Validation for Identifying Patients with CO Poisoning

Between January 2006 and August 2022, a total of 1991 patients visited our institution at least once due to CO poisoning (Code T58). Following a thorough review, 1920 cases were confirmed as CO poisoning. The PPV was determined to be 96.4% (95% confidence intervals [CI], 95.5–97.2%), demonstrating the accuracy of our methodology to identify CO poisoning patients from the NHIS data.

### 3.2. Baseline Characteristics of the Study Population

In total, 42,874 patients in the CO poisoning group (mean ± standard deviation [SD] age, 51.49 [16.6] years; females, 18,992 [44.3%]) and 905,285 individuals in the control group (age, 50.9 [16.3] years; females, 401,350 [44.33%]) were analyzed (Table 1). The assessment of covariate balance upon applying inverse probability weighting suggested that the covariates were well balanced. In addition, we confirmed that the proportional hazards assumption was not violated significantly for all target outcomes, as the Schoenfeld *p*-values were >0.05. The baseline demographic and general health characteristics of each cohort are summarized in Table 1. The mean (± SD) follow-up times for the CO poisoning and control cohorts were 2350.91 ± 1594.28 and 2438.99 ± 1609.39 d, respectively.

### 3.3. Development of Internal Malignancies

**Figure 1:**
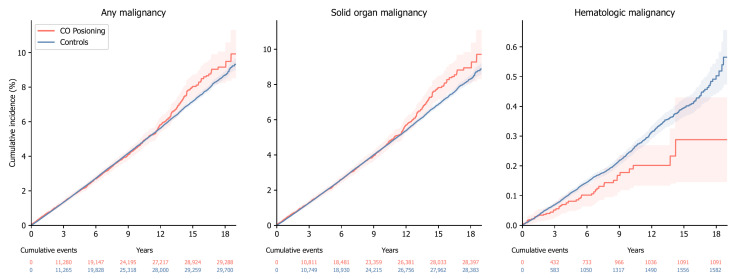
The cumulative incidence plot for malignancy. The cumulative incidence plot shows the cumulative incidence functions and the number of events in patients with carbon monoxide (CO) poisoning and controls. The shaded area shows the 95% confidence interval of the cumulative incidence functions.

Figure 1 illustrates the cumulative incidence plots for any malignancy, including solid organ and hematologic malignancies, in the CO poisoning and control cohorts. Incidence rates of any malignancies per 100,000 person-years were 50.55 (1370 malignancies for 271,005 person-years) for CO poisoning and 47.37 (28,341 malignancies for 5,983,418 person-years) for the controls (log-rank *p* < 0.001). Incidence rates for solid organ malignancies were 49.02 per 100,000 person-years (1329 cases in 271,118 person-years) for CO poisoning, compared with 45.23 per 100,000 person-years (27,081 cases in 5,987,275 person-years) for the controls (log-rank *p* < 0.001). For hematologic malignancies, incidence rates were 1.81 per 100,000 person-years (50 cases in 275,806 person-years) for CO poisoning and 2.49 per 100,000 person-years (1513 cases in 6,077,895 person-years) for the controls (log-rank *p* < 0.001).

**Figure 2:**
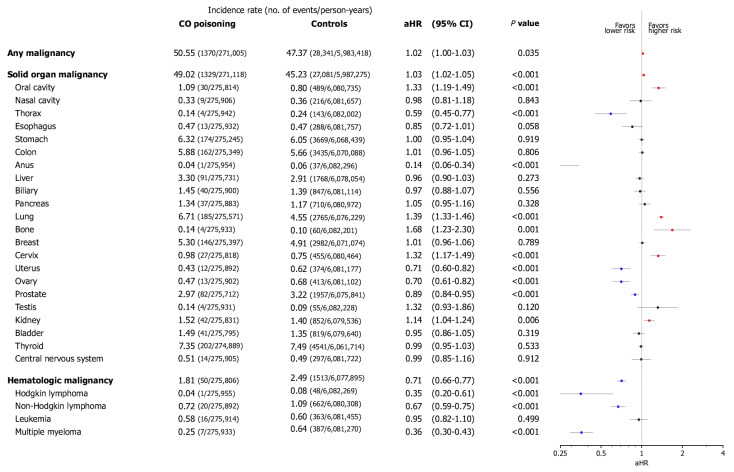
The risk of malignancy development related to carbon monoxide (CO) poisoning. The forest plot illustrates the adjusted hazard ratio (aHR) and 95% confidence intervals (CI) for each outcome among CO poisoning patients and the controls throughout the study period. The incidence rate is shown as the number of occurrences per 100,000 person-years. The plot presents the statistical estimates from the multivariable Cox proportional hazard analysis, in which covariates potentially associated with disease outcomes were selected based on the previous literature and biological plausibility, then balanced between the two cohorts using the inverse probability of treatment weighting and applied to adjust the multivariable models. The colored dots indicate statistically significant risk changes: red represents a statistically significant increase in risk, while blue represents a statistically significant decrease in risk.

**Figure 3:**
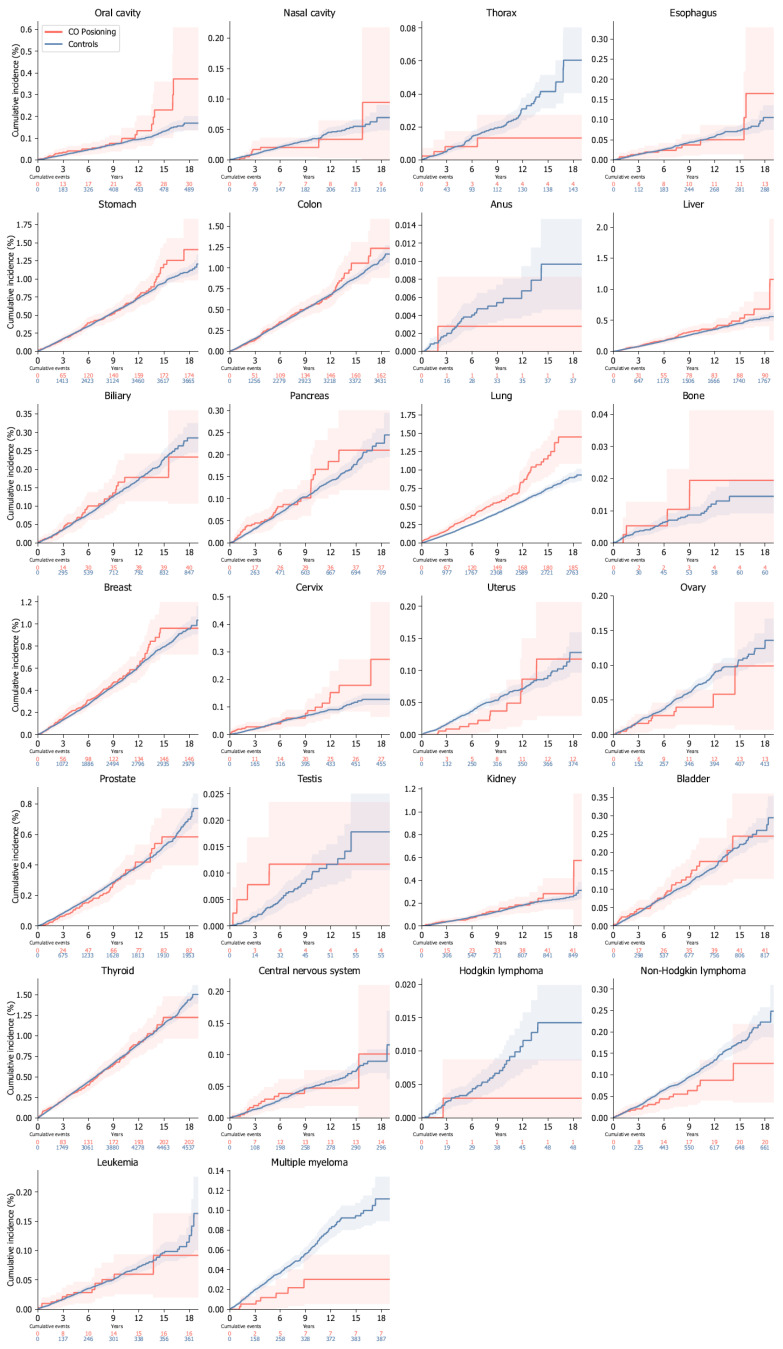
Individual cumulative incidence plots for internal malignancy by specific organ system. The cumulative incidence plot shows the cumulative incidence functions and the number of events in patients with carbon monoxide poisoning and the controls. The shaded area shows the 95% confidence intervals of the cumulative incidence functions.

Patients with CO poisoning had a significantly higher overall risk of internal malignancies than the controls (adjusted hazard ratios [aHR], 1.02; 95% CI, 1.00–1.03, *p* < 0.05) (Figure 2). Analyzing internal malignancies by categorizing them into solid organ and hematologic malignancies, patients with CO poisoning had a higher overall risk of solid organ malignancies than the controls (aHR, 1.03; 95% CI, 1.02–1.05, *p* < 0.001), whereas the risk of hematologic malignancies was lower (aHR, 0.71; 95% CI, 0.66–0.77, *p* < 0.001). The risk of organ-specific internal malignancy was elevated in the oral cavity (aHR, 1.33; 95% CI, 1.19–1.49), lungs (aHR, 1.39; 95% CI, 1.33–1.46), bones (aHR, 1.68; 95% CI, 1.23–2.30), cervix (aHR, 1.32; 95% CI, 1.17–1.49), and kidneys (aHR, 1.14; 95% CI, 1.04–1.24). Conversely, the risk of organ-specific internal malignancy was reduced in the thorax (aHR, 0.59; 95% CI, 0.45–0.77), anus (aHR, 0.14; 95% CI, 0.06–0.34), uterus (aHR, 0.71; 95% CI, 0.60–0.82), ovaries (aHR, 0.89; 95% CI, 0.84–095), Hodgkin lymphoma (aHR, 0.35; 95% CI, 0.20–0.61), non-Hodgkin lymphoma (aHR, 0.67; 95% CI, 0.59–0.75), and multiple myeloma (aHR, 0.36; 95% CI, 0.30–0.43). Figure 3 presents the cumulative incidence of organ-specific internal malignancies.

### 3.4. Subgroup Analyses

**Figure 4:**
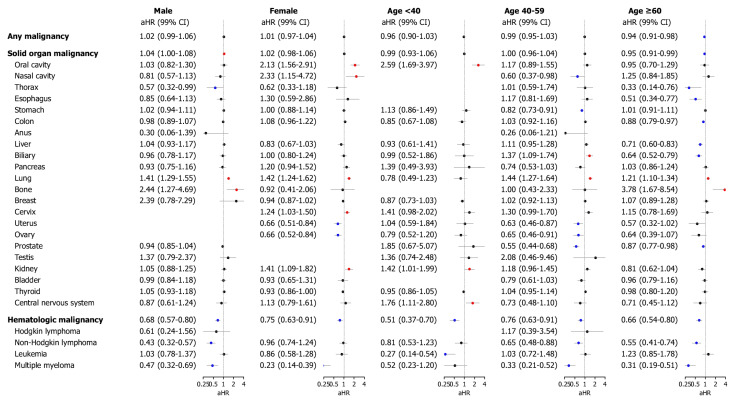
The malignancy development risk associated with carbon monoxide (CO) poisoning stratified based on sex and age. The subgroup analyses were performed according to sex and age. The forest plot shows the adjusted hazard ratio (aHR) and 95% confidence intervals (CI) for each outcome in patients with CO poisoning and the controls in each stratum. The plot presents the statistical estimates from the multivariable Cox proportional hazard analysis, in which covariates potentially associated with disease outcomes were selected based on the previous literature and biological plausibility, balanced between the two cohorts using inverse probability of treatment weighting, and applied to adjust the multivariable models. The colored dots indicate statistically significant risk changes: red represents a statistically significant increase in risk, while blue represents a statistically significant decrease in risk.

Next, outcomes were subjected to subgroup analyses (Figure 4), comparing the CO poisoning and control cohorts, stratified by sex and age (<40, 40–59, and ≥60 years). The overall risk of internal malignancies showed no difference in the CO poisoning group regardless of sex and age range. However, males with CO poisoning had an increased overall risk of solid organ malignancies (aHR, 1.04; 95% CI, 1.00–1.08). The CO poisoning group had a lower incidence of hematologic malignancies than the control group across all subgroups stratified by sex and age.

Examining the risk of internal malignancies based on HBO_2_ therapy and ICU admission following acute CO poisoning, no difference was found in the overall risk of internal malignancies in the CO poisoning group, regardless of HBO_2_ therapy or ICU admission status (Supplementary Figure S1). Upon categorizing internal malignancies into solid organ and hematologic types, differences were found in the risk of solid organ malignancies in the no-HBO_2_ therapy group and the no-ICU-admission group. However, the CO poisoning group exhibited a lower risk of hematologic malignancies than the control group across all subgroups. Incidence rates and aHRs for organ-specific internal malignancies based on HBO_2_ therapy and ICU admission are detailed in Supplementary Figure S1.

## 4. Discussion

In this nationwide population-based cohort study, CO poisoning was associated with a 1.02-fold increase in the overall risk of malignancy. Categorizing malignancies into solid organ and hematologic types, the risk of solid organ malignancies increased by 1.03-fold, whereas the risk of hematologic malignancies decreased by 0.71-fold in the acute CO poisoning group compared to the control group.

Huang et al. [21] reported that females with CO poisoning had a lower risk of developing breast cancer than those without CO poisoning. However, our study, which included a larger population, found no reduction in breast cancer prevalence in the CO poisoning cohort when compared with that in the control group.

A few animal studies found that CO exposure could inhibit tumor growth. For example, Vítek et al. [22] demonstrated that CO exposure markedly suppressed the proliferation of human pancreatic cancer cells and doubled the survival rates of mice with subcutaneously implanted pancreatic cancer cells. However, our study did not detect a reduction in pancreatic cancer prevalence in the CO poisoning cohort compared with that in the control group. Nemeth et al. [23] reported that low doses of CO inhibited lung cancer progression by modulating myeloid cell/macrophage infiltration and phenotype within the tumor microenvironment.

Conversely, we found a high incidence of lung cancer in the CO poisoning group, consistent with recent findings [24]. We postulated that CO levels could be determinants for its biological actions and pharmacological properties, and the inhibitory effects of CO on tumor growth may occur at lower CO concentrations [25,26,27]. Recently, we reported that CO poisoning could lead to the development of emphysematous changes in the lungs of a rat model [28]. Furthermore, smoking, a well-known carcinogen [29], increases the risk of respiratory diseases [30], cardiovascular diseases [31], and various types of malignancies [32,33,34]. Cigarette smoke-induced inflammation plays a key role in multiple pathologies by stimulating the release of pro-inflammatory cytokines [35]. Chronic CO exposure can induce epigenetic changes that regulate the specificity and duration of gene transcription [36].

Interestingly, our study revealed that patients with CO poisoning exhibited a lower incidence of hematologic malignancies than control participants, and this trend was also observed in most subgroup analyses. CO has been reported to impact the bone marrow system. According to one study, CO-mediated signaling induces a homeostatic program in stem cells that regulates the balance between hematopoiesis and stem cell preservation, ultimately altering the inflammatory milieu in mice [37]. In another study, carbon monoxide-releasing molecule-3 (CORM-3) increased the osteogenic differentiation of rat bone marrow-derived mesenchymal stem cells in vitro [38]. Additionally, CORM-3 may have potential therapeutic applications in treating periodontal disease and other bone defect conditions [39]. In recent years, novel CORMs have been identified as potential anticancer treatments [40,41,42], such as CORM-2 for lymphoma and acute myeloid leukemia [43]. A recent study suggests that CO regulates the differentiation of bone marrow progenitor cells, positively influencing the differentiation of blood cells and potentially reducing the incidence of hematologic cancers [44]. However, these results warrant careful interpretation because no study has evaluated the causality of malignancy development through acute CO poisoning, and only one human study [21] has explored the association between CO poisoning and malignancy development. Further basic research is necessary to explore the mechanisms underlying this observation.

In the current study, the risk of hematologic malignancies differed across all subgroups, warranting additional investigations to clarify this issue. Comparing the CO poisoning group treated without HBO_2_ with the control group, patients with CO poisoning had a significantly increased risk of solid malignancies than controls. The effect of HBO_2_ on tumor development remains uncertain. Although most experimental and clinical studies suggest that HBO_2_ does not directly impact tumor growth, some studies have reported potential anti-tumor effects [45,46,47]. Future research should focus on the role of HBO_2_ in the development of malignancies. Comparing the non-ICU-treated CO poisoning group with the control group, patients with CO poisoning exhibited a significantly higher risk of solid malignancies than control participants, although no such difference was detected between the ICU-treated CO poisoning and control groups. Accordingly, these results warrant cautious interpretation because patients with severe CO poisoning who were admitted to the ICU died early and were excluded from the analysis.

Our study had some limitations. First, while we verified our criteria for identifying patients diagnosed with CO poisoning using ICD-10 codes, the possibility of misclassification cannot be ruled out. Second, this was a retrospective observational cohort study; hence, causality cannot be established. Third, we were unable to determine the specifics of CO exposure, as this information was not available in the database. Fourth, despite balancing covariates such as demographic characteristics, income levels, and comorbidities, the possibility of residual confounding remains. Lastly, the small number of events for rare outcomes, such as Hodgkin lymphoma, limits the generalizability and robustness of our findings.

Despite these limitations, our study had several strengths. First, this study included a large, nationally representative Korean population. Moreover, the association between CO poisoning and the overall risk of developing malignancies, including organ-specific malignancies, has rarely been examined. Second, we also verified the diagnostic codes through our institution’s registry. Third, we used IPTW to balance multiple covariates that could impact the risk of cancer development.

## 5. Conclusions

In this nationwide cohort study, CO poisoning was associated with the development of internal malignancies. The risk of solid organ malignancies increased, whereas that of hematologic malignancies decreased. The long-term management of survivors of acute CO poisoning may consider monitoring for internal malignancies. Further studies are necessary to confirm these findings, explore the underlying mechanisms, and address the potential influence of residual confounding.

## Figures and Tables

**Table 1:** Demographic and clinical characteristics of the two cohorts before and after inverse probability treatment weighting.

Characteristics	Pre-Weighting, Patients, No. (%)			Post-Weighting, (Weighted %)		
	CO Poisoning (*n* = 42,874)	Control (*n* = 905,285)	SMD	CO Poisoning	Control	SMD
Age, mean ± SD, y	51.49 ± 16.60	50.86 ± 16.31	0.038	51.01 ± 78.14	50.89 ± 16.70	0.001
Sex, *n* (%)			0.001			0.009
Male	23,882 (55.70%)	503,935 (55.67%)		(55.20)	(55.67)	
Female	18,992 (44.30%)	401,350 (44.33%)		(44.80)	(44.33)	
Insurance type, *n* (%)			0.214			0.000
Standard	37,838 (88.25%)	853.379 (94.27%)		(94.02)	(93.99)	
Medicaid	5036 (11.75%)	51,906 (5.73%)		(5.98)	(6.01)	
Income level quartile, *n* (%)		-	0.233			0.000
Highest	9435 (22.01%)	267,512 (29.55%)		(28.55)	(29.24)	
Higher	12,754 (29.75%)	295,769 (32.67%)		(32.52)	(32.54)	
Lower	13,780 (32.14%)	242,736 (26.81%)		(28.65)	(26.96)	
Lowest	6905 (16.11%)	99,268 (10.97%)		(10.28)	(11.26)	
Area of residence, *n* (%)			0.118			0.002
Urban area	25,627 (59.77%)	488,085 (53.92%)		(54.28)	(54.18)	
Rural area	17,247 (40.23%)	417,200 (46.08%)		(45.72)	(45.82)	
Preexisting comorbidities, *n* (%)						
Hypertension	10,193 (23.77%)	196,103 (21.66%)	0.050	(21.93)	(21.76)	0.004
Diabetes mellitus	7513 (17.52%)	133,331 (14.73%)	0.076	(14.88)	(14.85)	0.001
Dyslipidemia	14,718 (34.33%)	273,248 (30.18%)	0.089	(30.34)	(30.37)	0.001
Chronic obstructive pulmonary disease	1181 (2.75%)	17,722 (1.96%)	0.053	(2.05)	(1.99)	0.004
Chronic heart failure	928 (2.16%)	16,827 (1.86%)	0.022	(1.91)	(1.87)	0.002
Liver cirrhosis	358 (0.84%)	5395 (0.60%)	0.028	(0.59)	(0.61)	0.002
Chronic kidney disease	268 (0.63%)	5970 (0.66%)	0.004	(0.70)	(0.66)	0.005
Lifestyle factors, *n* (%)						
Current smoker	8942 (20.86%)	176,448 (19.49%)	0.034	(19.06)	(19.55)	0.012
Drinking	26,408 (61.59%)	581,713 (64.26%)	0.055	(64.61)	(64.14)	0.011
General health examination data, mean ± SD						
Height, cm	165.11 ± 9.63	165.46 ± 9.54	0.036	165.3 ± 45.22	165.4 ± 9.77	0.012
Weight, kg	66.79 ± 14.28	67.13 ± 14.11	0.024	67.10 ± 67.74	67.12 ± 14.44	0.001
Waist circumference, cm	82.04 ± 10.70	81.92 ± 10.64	0.011	81.96 ± 50.48	81.93 ± 10.88	0.003
Blood pressure, mmHg						
Systolic	123.39 ± 15.23	123.54 ± 15.08	0.001	123.6 ± 71.74	123.5 ± 15.43	0.002
Diastolic	76.31 ± 10.46	76.20 ± 10.43	0.011	76.16 ± 49.10	76.20 ± 10.67	0.004
Laboratory values, mean ± SD						
Hemoglobin, g/dL	14.29 ± 1.67	14.28 ± 1.62	0.006	14.27 ± 7.83	14.28 ± 1.66	0.008
Fasting glucose, mg/dL	102.89 ± 29.08	101.87 ± 26.19	0.037	101.9 ± 127.50	101.9 ± 26.90	0.000
Aspartate aminotransferase, U/L	27.71 ± 18.82	26.96 ± 16.80	0.042	27.00 ± 77.98	26.99 ± 17.33	0.000
Alanine transaminase, U/L	27.40 ± 24.04	26.98 ± 22.62	0.018	26.98 ± 109.10	27.00 ± 23.19	0.001
Creatinine, mg/dL	0.87 ± 0.41	0.86 ± 0.43	0.002	0.87 ± 2.19	0.87 ± 0.42	0.004
CCI at index date, mean ± SD	1.41 ± 1.95	1.28 ± 1.90	0.068	1.31 ± 8.85	1.28 ± 1.95	0.011

CO = carbon monoxide, SMD = standardized mean difference, SD = standard deviation, CCI = Charlson comorbidity index.

## Data Availability

The data supporting the findings of this study were available from the National Health Insurance Service of Korea. Access restrictions, however, apply, as these data were used under license for the current study and are not publicly available. However, data will be made available by the authors upon reasonable request and with permission from the National Health Insurance Service of Korea.

## Supplementary Materials

The following supporting information can be downloaded at https://www.mdpi.com/article/10.3390/jcm14030937/s1, Figure S1: Malignancy development risk associated with CO poisoning, stratified by HBO2 therapy and ICU admission history during treatment.; Table S1: The international statistical classification of diseases, tenth revision (ICD-10) codes of the included diseases.
