# Supplementary material for: Internal Malignancy Risk After Carbon Monoxide Poisoning: A Nationwide Population-Based Cohort Study
Source: J Clin Med. 2025 Jan 31;14(3):937. doi: 10.3390/jcm14030937 (PMC11818198; doi:10.3390/jcm14030937)
Supplement: Supplementary file 1 [file jcm-14-00937-s001.zip › jcm-3344856-supplementary.pdf]

Supplementary materials for:

Internal malignancy risk after carbon monoxide poisoning : a nationwide population based cohort study

Gyo Jin Ahn, Solam Lee, Seok Jeong Lee, Yong Sung Cha

Table of Contents:

A. Supplementary Figures

Supplementary Figure S1. Malignancy development risk associated with CO poisoning, stratified by HBO2 therapy and ICU admission history during treatment.

B. Supplementary Tables

Supplementary Table S1. International statistical classification of diseases, tenth revision (ICD-10) codes of the included diseases

Supplementary Figure S1. Malignancy development risk associated with CO poisoning, stratified by HBO_2 therapy and ICU admission history during treatment.

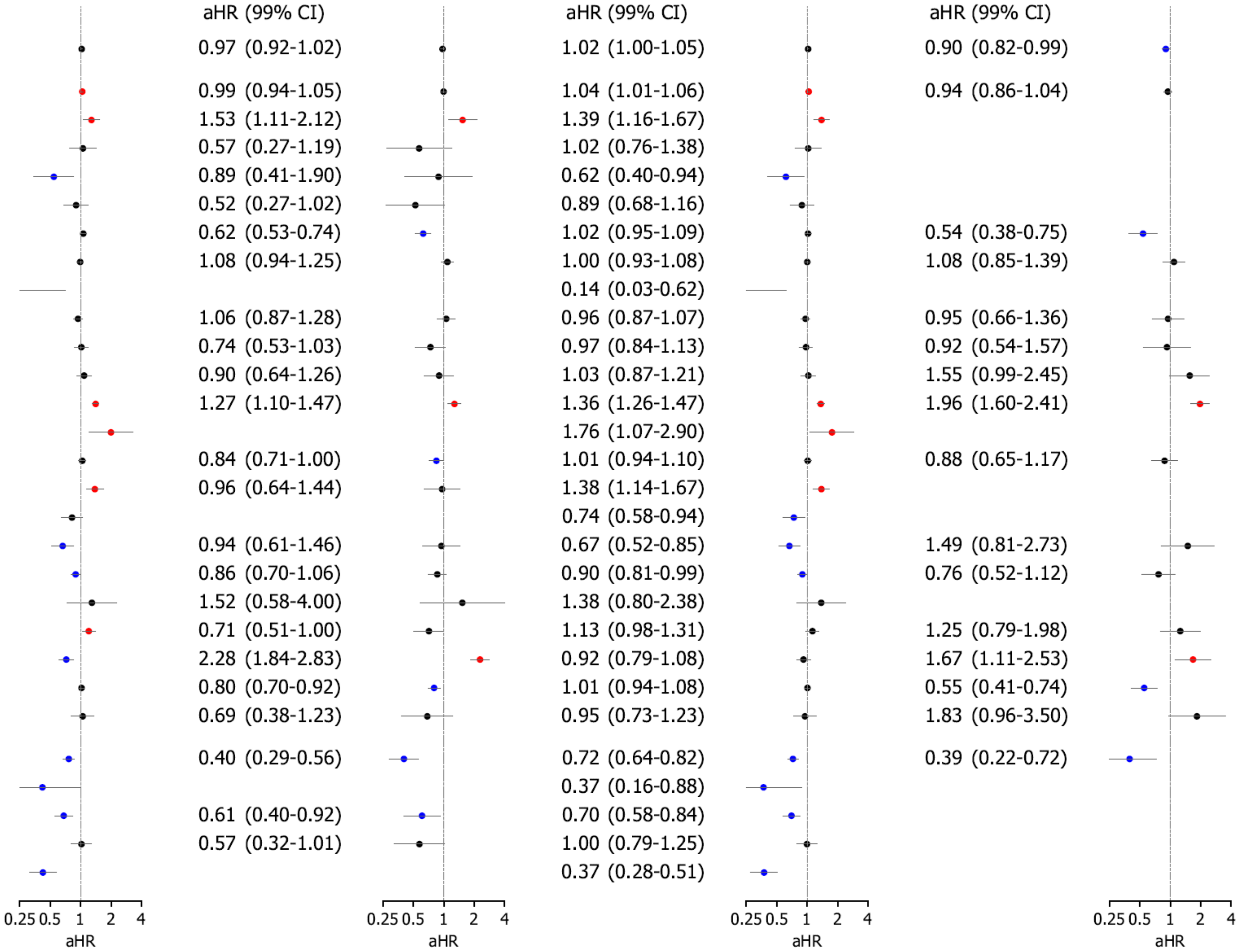

	HBO_2 No aHR (99% CI)	HBO_2 Yes aHR (99% CI)	ICU No aHR (99% CI)	ICU Yes aHR (99% CI)
**Any malignancy**	1.03 (1.00-1.05)	0.97 (0.92-1.02)	1.02 (1.00-1.05)	0.90 (0.82-0.99)
**Solid organ malignancy**	1.04 (1.01-1.07)	0.99 (0.94-1.05)	1.04 (1.01-1.06)	0.94 (0.86-1.04)
Oral cavity	1.28 (1.06-1.55)	1.53 (1.11-2.12)	1.39 (1.16-1.67)	
Nasal cavity	1.05 (0.78-1.43)	0.57 (0.27-1.19)	1.02 (0.76-1.38)	
Thorax	0.54 (0.34-0.86)	0.89 (0.41-1.90)	0.62 (0.40-0.94)	
Esophagus	0.90 (0.69-1.19)	0.52 (0.27-1.02)	0.89 (0.68-1.16)	
Stomach	1.06 (0.99-1.14)	0.62 (0.53-0.74)	1.02 (0.95-1.09)	0.54 (0.38-0.75)
Colon	0.99 (0.92-1.07)	1.08 (0.94-1.25)	1.00 (0.93-1.08)	1.08 (0.85-1.39)
Anus	0.16 (0.04-0.70)		0.14 (0.03-0.62)	
Liver	0.94 (0.85-1.05)	1.06 (0.87-1.28)	0.96 (0.87-1.07)	0.95 (0.66-1.36)
Biliary	1.02 (0.87-1.19)	0.74 (0.53-1.03)	0.97 (0.84-1.13)	0.92 (0.54-1.57)
Pancreas	1.08 (0.92-1.28)	0.90 (0.64-1.26)	1.03 (0.87-1.21)	1.55 (0.99-2.45)
Lung	1.41 (1.30-1.53)	1.27 (1.10-1.47)	1.36 (1.26-1.47)	1.96 (1.60-2.41)
Bone	2.00 (1.22-3.29)		1.76 (1.07-2.90)	
Breast	1.04 (0.96-1.13)	0.84 (0.71-1.00)	1.01 (0.94-1.10)	0.88 (0.65-1.17)
Cervix	1.38 (1.14-1.68)	0.96 (0.64-1.44)	1.38 (1.14-1.67)	
Uterus	0.82 (0.64-1.05)		0.74 (0.58-0.94)	
Ovary	0.66 (0.52-0.85)	0.94 (0.61-1.46)	0.67 (0.52-0.85)	1.49 (0.81-2.73)
Prostate	0.89 (0.80-0.99)	0.86 (0.70-1.06)	0.90 (0.81-0.99)	0.76 (0.52-1.12)
Testis	1.29 (0.73-2.30)	1.52 (0.58-4.00)	1.38 (0.80-2.38)	
Kidney	1.20 (1.04-1.40)	0.71 (0.51-1.00)	1.13 (0.98-1.31)	1.25 (0.79-1.98)
Bladder	0.72 (0.61-0.86)	2.28 (1.84-2.83)	0.92 (0.79-1.08)	1.67 (1.11-2.53)
Thyroid	1.02 (0.96-1.09)	0.80 (0.70-0.92)	1.01 (0.94-1.08)	0.55 (0.41-0.74)
Central nervous system	1.05 (0.81-1.36)	0.69 (0.38-1.23)	0.95 (0.73-1.23)	1.83 (0.96-3.50)
**Hematologic malignancy**	0.76 (0.67-0.87)	0.40 (0.29-0.56)	0.72 (0.64-0.82)	0.39 (0.22-0.72)
Hodgkin lymphoma	0.42 (0.18-1.00)		0.37 (0.16-0.88)	
Non-Hodgkin lymphoma	0.68 (0.56-0.83)	0.61 (0.40-0.92)	0.70 (0.58-0.84)	
Leukemia	1.02 (0.81-1.29)	0.57 (0.32-1.01)	1.00 (0.79-1.25)	
Multiple myeloma	0.42 (0.31-0.57)		0.37 (0.28-0.51)	

The subgroup analyses were performed according to hyperbaric oxygen (HBO_2) therapy and intensive care unit (ICU) admission history during carbon monoxide (CO) treatment. The forest plot shows the adjusted hazard ratio (aHR) and 95% confidence interval (CI) for each outcome in patients with CO poisoning and controls in each stratum. The plot presents the statistical estimates from the multivariable Cox proportional hazard analysis in which covariates potentially associated with disease outcomes were selected based on previous literature and biological plausibility, balanced between the two cohorts using inverse probability of treatment weighting (IPTW), and applied to adjust the multivariable models. The colored dots indicate statistically significant risk changes: red represents a statistically significant increase in risk, while blue represents a statistically significant decrease in risk.

Supplementary table S1: International statistical classification of diseases, tenth revision (ICD-10) codes of the included diseases.

Disease	ICD-10 code
**Solid Organ Malignancy**	
Oral Cavity & Pharynx	C01, C02, C03, C04, C05, C06, C07, C08, C09, C10, C11, C12, C13, C14
Nasal Cavity & Larynx	C30, C31, C32, C33
Thorax	C37, C38, C39, C45
Esophagus	C15
Stomach	C16
Colon & Rectum	C18, C19, C20
Anus	C21
Liver	C22
Gallbladder & Biliary tract	C23, C24
Pancreas	C25
Lung	C34
Bone	C40, C41
Breast (Female)	C50
Cervix (Female)	C51, C52, C53
Uterus (Female)	C54, C55
Ovary (Female)	C56
Prostate (Male)	C61
Testis (Male)	C62
Kidney	C64, C65
Bladder	C66, C67
Thyroid	C73
**Hematologic Malignancy**	
Central Nervous System	C70, C71, C72
Hodgkin lymphoma	C81
Non-Hodgkin lymphoma	C82, C83, C84, C85, C86
Leukemia	C91, C92, C93, C94, C95
Multiple myeloma	C88, C90
